# Tissue, developmental, and caste-specific expression of odorant binding proteins in a eusocial insect, the red imported fire ant, *Solenopsis invicta*

**DOI:** 10.1038/srep35452

**Published:** 2016-10-21

**Authors:** Wei Zhang, Arun Wanchoo, Almudena Ortiz-Urquiza, Yuxian Xia, Nemat O. Keyhani

**Affiliations:** 1 Genetic Engineering Research Center, School of Life Sciences, Chongqing University, Chongqing 400045, PR China; 2 Department of Microbiology and Cell Science, Institute of Food and Agricultural Sciences, University of Florida, Gainesville, FL, 32611, USA

## Abstract

Insects interact with the surrounding environment via chemoreception, and in social insects such as ants, chemoreception functions to mediate diverse behaviors including food acquisition, self/non-self recognition, and intraspecific communication. The invasive red imported fire ant, *Solenopsis invicta*, has spread worldwide, displaying a remarkable environmental adaptability. Odorant binding proteins (OBPs) are chemical compound carriers, involved in diverse physiological processes including odor detection and chemical transport. *S. invicta* contains a highly divergent 17-member OBP gene family, that includes an ant-specific expansion and the social organization implicated *Gp-9* (*OBP3*) gene. A systematic gene expression analysis of the *SiOBP* repertoire was performed across social caste (workers, male and female alates), tissues (antennae, head, thorax, and abdomen), and developmental stages (egg, larvae, and pupae), revealing that although *SiOBP*s were expressed in the antennae, the major regions of expression were in the head and thorax across all castes, and the abdomen in male and female alates. *SiOBP*s were very highly expressed in female alates and at somewhat lower levels in male alates and workers. *SiOBP*s were differentially expressed, with unique signatures in various castes and tissues, suggesting functionality of SiOBPs beyond olfaction Expression patterns of *SiOBP* subgroups also showed relationships with their evolutionary relatedness.

Olfaction underlies significant aspects of individual and social behaviors in insects. Insect olfaction is mediated by a subset of specialized cuticular structures known as sensilla, most commonly found on the antennae, although sensilla can be found on other body structures as well, i.e. the maxillary palp and at the ends of various appendages^1^,^2^. Olfactory sensilla contain olfactory sensory neurons (OSNs), suspended in an aqueous lymph, that in turn harbor olfactory receptors (ORs) in their dendritic membranes. While ORs are capable of being directly affected by chemical compounds^3^, many odorants are hydrophobic and/or have low aqueous solubility, necessitating a means of delivering odorants to ORs or other receptors. Within this context, several classes of chemosensory ligand carrier proteins have been described, one of the major being the family of odorant binding proteins (OBPs; these proteins do not share homology to the similarly named vertebrate OBPs)^4^. OBPs are small molecular weight (~130–150 amino acids), soluble, ligand carrier proteins and include the pheromone binding proteins (PBPs) and the general odorant binding proteins (GOBPs). OBPs have also been classified into three groupings; classic, C-minus, and C-plus, based on the distribution and number of cysteine residues. Insect OBPs exist as gene families and a significant number have been identified either via genome sequencing and/or expression (e.g. RNA_seq) analyses, particularly those focused on the antennal transcriptome^5^,^6^,^7^. Genomic and transcriptomics analyses in several ant species strongly support the idea that both OBPs and the related chemosensory proteins (CSPs) are expressed in the antennae, however many of these proteins appear to be expressed primarily in non-chemosensory tissues^8^. Thus, while a number of OBPs have been linked to olfaction, the roles of many OBPs remain obscure and they are likely to play important roles as chemical carriers in a wide range of physiological processes^9^.

Ants are eusocial organisms having evolved a complex level of communal organization highlighted by specialization of labor and reproduction. Ant societies have been referred to as “super-organisms”, i.e. a group of cooperating animals resulting from “the combined operation of tiny and short-lived minds” leading to a colony that functions, on some levels, as a single organism^10^. The red imported fire ant, *Solenopsis invicta* forms complex societies that are territorial and whose members display strong nest-mate recognition, elaborate task specialization, and a multi-tiered caste system^11^. Workers represent a terminal differentiated form, and reproductives, i.e. winged male and female alates are produced seasonally as the colony matures, a process that can take up to five years. Depending upon their genetic background *S. invicta* colonies can have one (monogyny) or multiple queens (polygyny)^12^. These two differing colony organizations extend to important differences in the biology and responses of *S. invicta*, and early genetic investigations implicated variation at the *Gp-9* gene as being linked to the regulation of these divergent social behaviors^13^,^14^. *Gp-9* overexpression has also been associated with fire ant workers^15^, and the gene has subsequently been shown to be an odorant binding protein (SiOBP3)^16^ and its role in mediating social organization has been challenged^17^,^18^. As the genome of *S. invicta* was described, a 13 Mb “Y-like social chromosomal” fragment (~55% of the chromosome) that includes the *Gp-9/SiOBP3* gene was found^19^,^20^. Recombination appears suppressed within this region that contains at least 616 open reading frames, and its rearrangement correlates with the divergent social phenotypes. Thus *Gp-9/SiOBP3* may form one protein product among many that contribute to certain aspects of social behavior.

Analysis of the *S. invicta* genome has indicated the presence of 17 OBPs genes and one OBP pseudogene^16^. Here we have performed a systematic analysis of the expression of the 16 *S. invicta* OBPs (no signal for *SiOBP17* was detected in any samples examined) across different tissues; antennae, head, thorax, and abdomen, castes; workers, and male and female alates, and developmental stages; eggs, larvae, and pupae. These data revealed that *SiOBP*s are generally more highly expressed in head and thorax tissues than in the antennae of either workers or male and female alates. *SiOBP* expression was also abundant in the abdomen of male and female alates but low in the abdomen of workers. *SiOBP*s were also differentially expressed throughout all of the developmental stages examined. *SiOBP3* (*Gp-9*) was highly expressed in female alates (head, thorax, and abdomen but lower in the antennae), in worker heads, and to a somewhat lower extent in male alates (head, thorax, abdomen > antennae. Our data provide a systematic overview of OBP expression in a social insect, suggesting their broad participation in developmental and physiological processes beyond olfaction, allowing for further functional probing of their activities.

## Results

### The odorant binding protein repertoire of *Solenopsis invicta*

**Figure 1:**
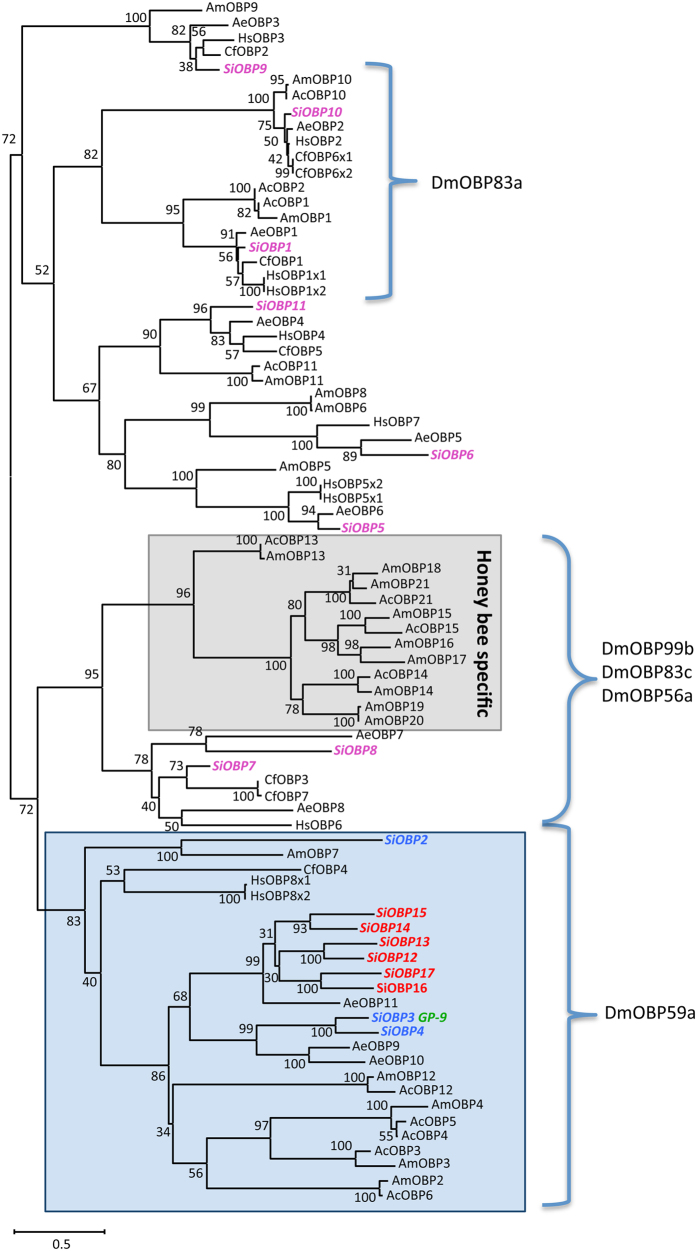
Phylogenetic analyses of *S. invicta* chemosensory proteins (SiOBPs). Limited phylogenetic analyses of *S. invicta* OBPs compared to the OBP repertoires found in the ant species, *A. echinatior* (AeOBP), *C. floridanus* (CfOBP), and *H. saltator* (HsOBP), and the honey bees, *A. mellifera* (AmOBP) and *A. cerana* (AcOBP) (Genbank accession numbers given in Supplemental Table S4). The closest *D. melanogaster* homologs genetically characterized are shown after brackets. Numbers at nodes indicate bootstrap values. The tree is midpoint-rooted in the absence of a suitable out-group.

A set of 17 *OBP* genes has been previously identified in the *S. invicta* genome (Supplemental Table S1). A limited phylogenetic tree of deduced protein sequences that includes OBPs from the available ant genomes; i.e. *Acromrymex echinatior* (New World, fungus growing, leaf cutter ant), *Camponatus floridanus* (carpenter ant), and *Harpegnathos saltator* (Indian/Jerdon jumping ant), two honey bee species; *Apis mellifera* (European honeybee) and *Apis cerana* (Asiatic honeybee), illustrates the division of the SiOBPs into two discrete major branches (Fig. 1, related *Drosophila melanogaster* OBPs are also shown). SiOBPs 1, 5, 6, 9, 10, and 11 were distributed with various orthologous OBPs from different insects (top clade in figure). On the other branch (bottom in figure), two discrete clusters could be discerned; one containing SiOBPs 7 and 8, along with other ant orthologs (e.g. AeOBP7 and CfOBPs 3 and 7) that were distinct from an apparent honey bee OBP gene expansion. In the second cluster (lower part of figure) SiOBP2 appeared distinct and distributed with orthologous sequences from the honeybee (*A. mellifera* OBP7). The remaining SiOBPs; 3 & 4, and 12 through 17, formed closely grouped members, respectively. SiOBPs 12–17 form a *S. invicta* OBP gene expansion as has been previously reported^16^, along with an ortholog identified in *A. echinatior* (AeOBP11).

**Figure 2:**
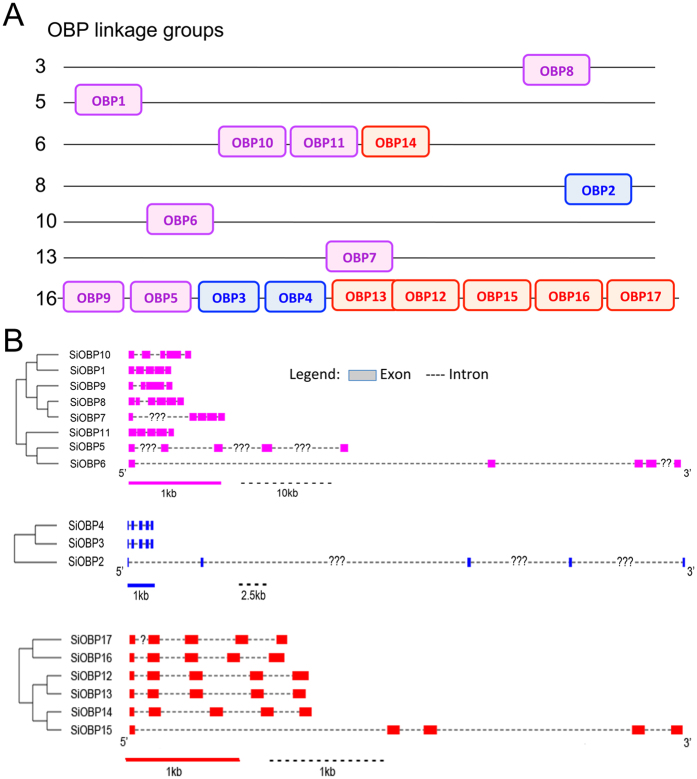
(**A**) Mapping of *SiOBP*s to genomic contigs. *SiOBP*s positions are not drawn to scale but indicate approximate locations on the linkage groups. *SiOBP*s 12 and 13 were overlapping in genomic context (see text for explanation). *SiOBP* exon boxes are color coded to match their phylogenetic placement as in Fig. 1(**B**) Intron**-**exon structures of the *SiOBP*s.

A schematic of the genomic context of the *SiOBP*s revealed their distribution within 7 linkage groups (Fig. 2A, OBPs are color coded as in Fig. 1). *SiOBP*s were found on linkage groups as follows: LG-3, *SiOBP*8; LG-5, *SiOBP*1; LG-6, *SiOBP*s 10, 11, and 14; LG-8, *SiOBP*2; LG-10, *SiOBP*6; LG-13, *SiOBP*7; LG-16, *SiOBP*s 3–5, 9, 12, 13, and 15–17. This latter linkage group (LG-16) corresponds to the Y-like social chromosome implicated as controlling two divergent forms of social organization (monogyny versus polygyny) in *S. invicta*^19^). *SiOBP*s 12 and 13 were overlapping in their overall genomic contexts, with the *SiOBP*13 start site 5172 bp before that of *SiOBP*12. These OBPs also shared the last exon, with all others different, thus sequence corresponding the exonic region is found partially or totally in the intronic sequences of the other. Within the context of the *SiOBP*s, a correlation between phylogeny and intron/exon structure, as determined by the online Gene Structure Display Server (GSDS: http://gsds.cbi.pku.edu.cn) was noted (Fig. 2B). Members of the general *SiOBP*s (1, 5–11) could be further separated into two branches, one consisting of *SiOBP*s 1 and 7–10 and the other of *SiOBP*s 5, 6, and 11. All of former, with the exception of *SiOBP*7 whose length of the first intron could not be determined due to incomplete sequence information, were similar in exon/intron structure, having numerous small introns with an overall coding/non-coding length <1 kB. *SiOBP*11 was similar to the others as above, and three of the introns in *SiOBP*5 were indeterminate, thus precluding full analyses, although it did contain at least one intron ~5 kb in length. However, *SiOBP*6 was notably different than the others in this group, containing two large introns (15–38 kb), one indeterminate intron, and an overall sequence length of >60 kb. Of *SiOBP*s (i.e. 2, 3 and 4) found in the clade containing the ant-specific OBP gene expansion (but distinct from these, see Fig. 1), *SiOBP*s 3 and 4, were essentially identical in intron/exon structure (small intron, overall genomic sequence length ~ 1 kb), highly similar in sequence (69% identity), and tandemly arranged in linkage group 16 (Fig. 2A), strongly supporting a recent gene duplication event. In contrast, *SiOBP*2 contained at least three large (9–23.5 kb) introns, one indeterminate, and an overall genomic context of >50 kb. Members of the ant-specific *SiOBP* gene expansion (*SiOBP*s 12–17) were similar to each other (with the exception of *SiOBP*15) and distinct from the other *SiOBP*s. *SiOBP*s 12, 13, 14, and 16 contained introns 100–500 bp in length (somewhat larger than seen for the *SiOBP*s 3 and 4 described above) with overall genomic sequences of ~1.5 kb. *SiOBP*15 contained one intron of ~1.7 kb and another of indeterminate size, with an overall genomic context of >5 kb.

### Expression pattern of the *SiOBP*s in *S. invicta* workers

Oligonucleotide primers were designed and validated for quantitative RT-PCR as described in the Methods section. Primer efficiency, amplification of single bands corresponding to the predicted size of each amplicon was verified (Supplementary Tables S2 and S3). All amplicons were cloned and used to construct standard curves for absolute quantification of each respective transcript number in the samples. In addition to primers designed to the 17 *SiOBP*s, primers were also designed to 2 different housekeeping genes, glyceraldehyde-phosphate dehydrogenase (GAPDH) and elongation factor 1α (EF1α) for use as references. As significant variation of both genes was seen across tissues and life stages, absolute quantification of the expression of each gene was performed and the expression levels for GAPDH and EF1α are presented along with that for the *SiOBP*s. Normalization to the housekeeping genes was also performed in order to allow for better comparison between tissues in certain instances. The expression distribution of *SiOBPs* within each caste or developmental stage is included as supplemental materials (Supplemental Excel File S1). These data show representations of the tissue distribution for each *SiOBP* calculated separately for workers, male and female alates, and across developmental stages by dividing the absolute expression for each sample (cell in the heat map) by the total expression for that *SiOBP* in the caste or developmental stage (Supplemental Excel File S1, “Tissue distribution %” subtab). The original calculated data (# transcript copies/1 μg RNA) are also included.

**Figure 3:**
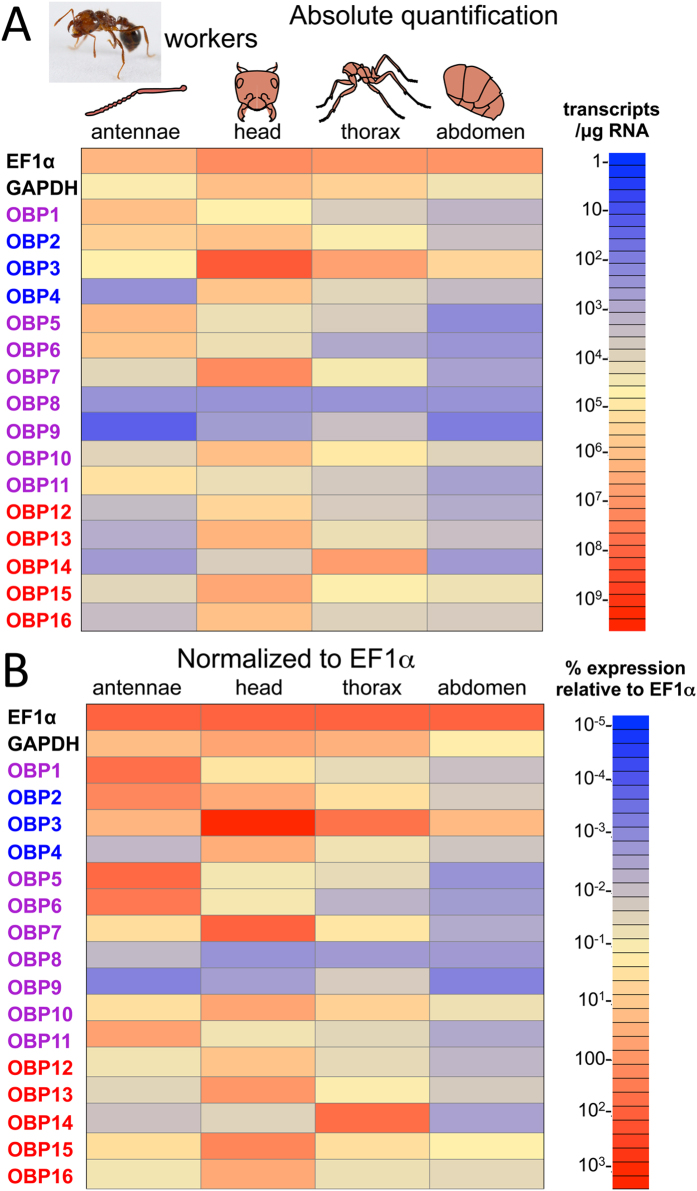
Expression of *SiOBP*s in *S. invicta* workers. (**A**) qRT-PCR was performed on RNA isolated from fire ant worker antennae, head, thorax, and abdomen. Absolute quantification values are shown. Color scale shows transcripts/μg total RNA used for cDNA synthesis. (**B**) Data in (**A**) normalized to EF1α expression levels.

*SiOBP*s gene expression was examined in dissected tissues derived from workers that included antennae, head, thorax, and abdomen (Fig. 3). *SiOBP*17 failed to yield any product including when tested against *S. invicta* genomic DNA and was eliminated from further analyses. In terms of absolute expression, *SiOBP* expression was highest in the head; with *SiOBP*3 highly expressed, followed by *SiOBP*7, and then *SiOBP*s 2, 4, 10, 12, 13,15, and 16 (Fig. 3A). Expression levels of *SiOBP*s 8 and 9 in the head were low, whereas moderate expression levels of the remaining *SiOBP*s (1, 5, 6, 11, and 14) were seen in these tissues. In general, expression of the *SiOBP*s in the antennae ranged from moderate (*SiOBP*s 1, 2, 5, 6, and 11 > *SiOBP*s 3, 7, 10, and 15) to low (*SiOBP*s 4, 8, 9, 12–14, and 16). *SiOBP*s 3 and 14 were highly expressed in the thorax, with moderate expression levels of the remaining OBPs observed, with the exception of *SiOBP*s 6, 8 (in particular), 9, 11, and 12, whose expression levels were lower than the others. *SiOBP*s, in particular OBPs 1, 2, 4–9, 11, 12, and 14, were apparently poorly expressed in the abdominal section, with only moderate levels of OBP3 seen in these tissues. When data are normalized to EF1α levels the general trends in each tissue remained consistent with the notable change of higher relative expression of the *SiOBP*s in the antennae (Fig. 3B). For the other tissues, normalization resulted in only minor changes in the *SiOBP* expression pattern.

### Expression pattern of the *SiOBP*s in *S. invicta* male and female alates

**Figure 4:**
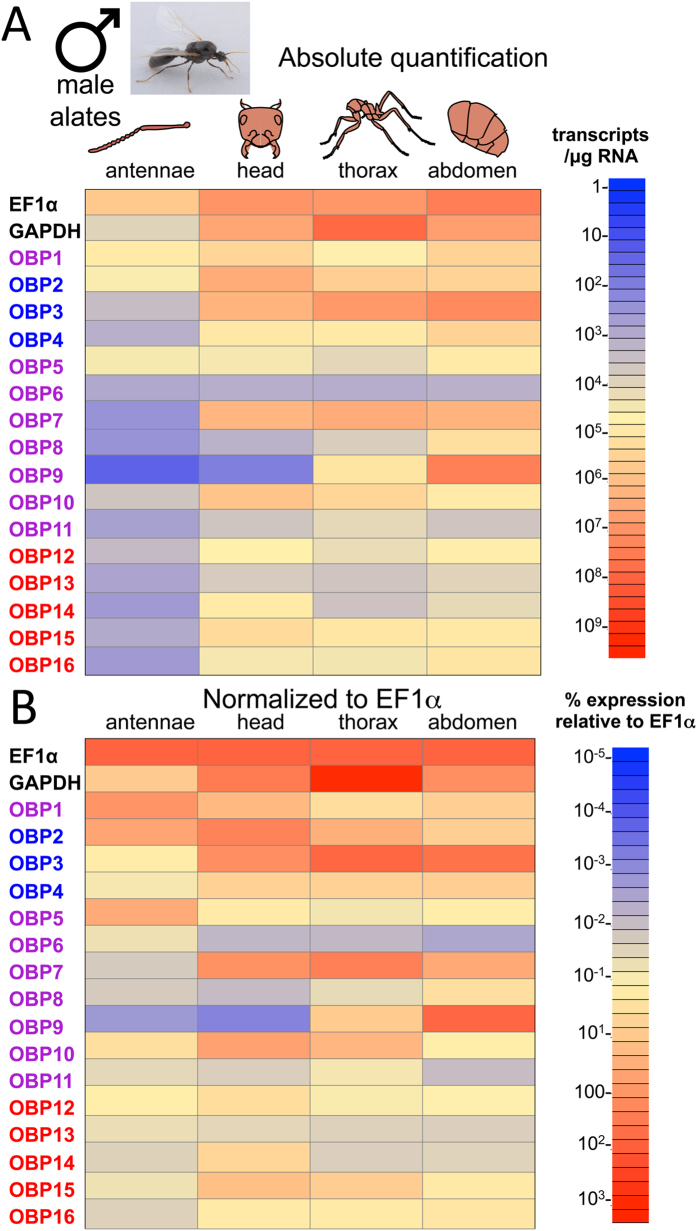
Expression of *SiOBP*s in *S. invicta* male alates. (**A**) qRT-PCR was performed on RNA isolated from fire ant male alate antennae, head, thorax, and abdomen. Absolute quantification values are shown. Color scale shows transcripts/μg total RNA used for cDNA synthesis. **(B)** Data in (**A**) normalized to EF1α expression levels.

In terms of absolute expression, *SiOBP*s were poorly expressed in the male antennae, with low transcript levels seen for *SiOBP*s 4, and 6–16, and only moderate expression levels were seen for *SiOBP*s 1–3, and 5 (Fig. 4A). In contrast, male head, thorax, and abdomen regions displayed higher levels of OBP expression. High levels of *SiOBP*s 1–3, 7 and 10 were noted for the male head, with moderate levels of expression seen for *SiOBP*s 4, 5, 12, 14, 15, and 16, low levels seen for *SiOBP*s 6, 8, 11, and 13, with only barely detected levels of *SiOBP*9 noted. *SiOBP*s 3 and 7 were highly expressed in the thorax, with moderate expression of *SiOBP*s 2, 9, and 10, with lower levels of *SiOBP*s 1, 4, 15, and 16 were seen in the male thorax, with *SiOBP*s 5, 6, 8, and 11 through 14 expressed at lower levels. *SiOBP*s were generally robustly expressed in the male abdomen, with the exception of *SiOBP*s 6, 8 and to a lower extent *SiOBP*s 13 and 14 whose expression levels were low. *SiOBP*s 3 and 9 were the most highly expressed, followed by *SiOBP*s 1, 2, 4, 5, 7, 8, 10, 12, 15 and 16. Normalization to EF1α levels had a moderate effect in increasing the apparent expression levels of the *SiOBP*s in the male antennae (Fig. 4B), although overall expression was lower than the other tissues. Only minor changes in *SiOBP* gene pattern expressions were seen for the other male tissues examined when normalized.

**Figure 5:**
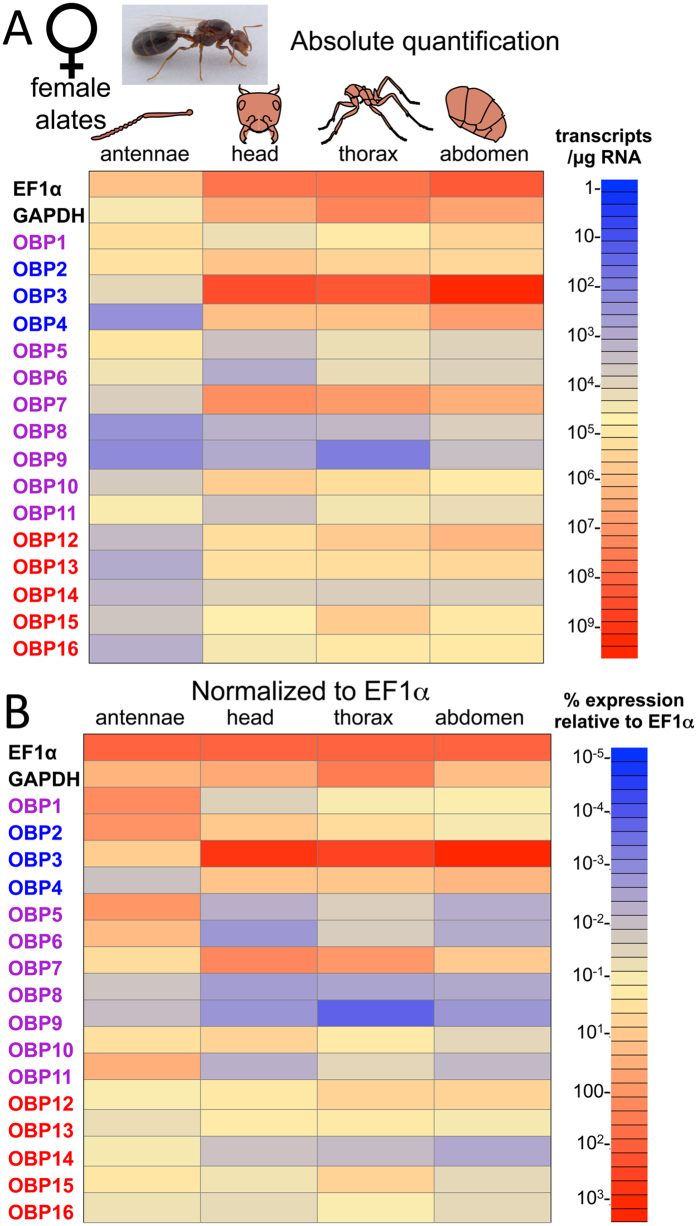
Expression of *SiOBP*s in *S. invicta* female alates. (**A**) qRT-PCR was performed on RNA isolated from fire ant female alate antennae, head, thorax, and abdomen. Absolute quantification values are shown. Color scale shows transcripts/μg total RNA used for cDNA synthesis. (**B**) Data in (**A**) normalized to EF1α expression levels.

Similar to male alates, absolute expression of the *SiOBP*s was lowest in the female antennae as compared to the other tissues (Fig. 5A), however, as seen in the male antennae EF1α levels were also lower in the female antennal tissues as compared to the others. Low expression levels of *SiOBP*s 3, 4, 7–10, 12–15 were seen in female antennae, whereas moderate expression of *SiOBP*s 6 and 11, and somewhat higher expression levels of *SiOBP*s 1, 2, and 5 were seen in these structures. In contrast very high levels of *SiOBP*3 and high levels of *SiOBP*s 2, 4, 7, 10, 12, and 13 were seen in the female alate head. The female alate head also showed moderate expression levels of *SiOBP*s 15 and 16, somewhat lower levels of expression of *SiOBP*s 1 and 14, and low levels of *SiOBP*s 5, 6, 8, 9 and 11 expression. *SiOBP* expression in the female alate thorax was similar to the head. Very high levels of *SiOBP*3 were observed, with high levels of expression of *SiOBP*s 2, 4, 7, 10, 12, 13, and 15, seen in the female alate thorax. Moderate to low levels of expression of *SiOBP*s 1, 5, 6, 11,14 and 16 were seen in these tissues. *SiOBP*s 8, and in particular 9, were expressed at low to very low levels in the female alate thorax. As seen in the head and thorax regions, *SiOBP*3 was highly expressed in the female alate abdomen. In addition, *SiOBP*s 1, 2, 4, 7, 12, and 13 were highly expressed in the female abdomen, with moderate expression of *SiOBP*s 10, 15, and 16, and low expression of *SiOBP*s 5, 6, 8, 9, and 14 seen in these tissues. Normalization to EF1α levels, increased the apparent expression levels of the *SiOBP*s in the female antennae, but had only minor effects with respect to the other tissues examined (Fig. 5B).

### Expression pattern of the *SiOBP*s in different developmental stages of *S. invicta*

**Figure 6:**
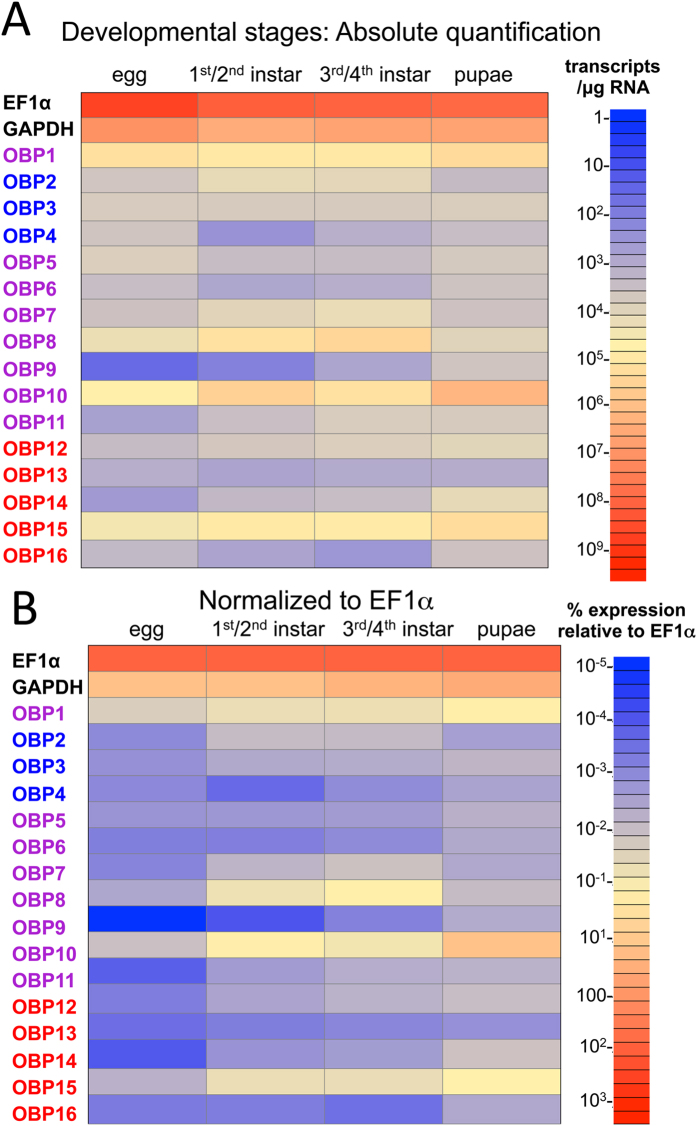
Developmental expression of *SiOBP*s. (**A**) qRT-PCR was performed on RNA isolated from fire ant eggs, early and late instar and pupae. Absolute quantification values are shown. Color scale shows transcripts/μg total RNA used for cDNA synthesis. (**B**) Data in (**A**) normalized to EF1α expression levels.

The expression pattern of the *SiOBP*s was examined in eggs, larvae, and pupae (Fig. 6). Larvae were further separated into early (1^st^ and 2^nd^) and late (3^rd^ and 4^th^) instars based on size. Absolute expression analyses indicated that *SiOBP*s 1, 8 10, and 15 were the most highly expressed OBPs in eggs, with *SiOBP*s 2 through 7, 12 and 16, less, but still moderately expressed (Fig. 6A). Compared to the preceding mentioned genes, lower expression of *SiOBP*s 11, 13, and 14 was seen in eggs, and very low expression of *SiOBP* 9 was observed in these samples. Early instar larvae displayed high to moderate levels of *SiOBP*s 1, 8, 10, and 15. In addition, moderate expression of *SiOBP*s 2, 3, 7, and 12, low expression of *SiOBP*s 5, 11, and 14, and very low expression of *SiOBP*s 4, 6, 13, and 9 were seen in the early instar larvae. In late larvae, expression of *SiOBP*s 1, 8, 10, and 15 was moderate to high, expression of *SiOBP*s 2, 3, 7, 11, and 12 was moderate, with moderate to low levels of *SiOBP*s 4, 6, 9, 13 and 16 seen. *SiOBP*s were also found in pupal samples. *SiOBP*s 1, 10, and 15 were highly expressed, with *SiOBP*s 3, 5, 8, 9, 11, 12, 14 and 16 moderately expressed, and *SiOBP*s 2, 4, 6, 7, and 13 moderate/low in expression. In these tissues, as compared to the adults, EF1α levels were significantly higher (P < 0.001) and normalization to EF1α resulted in very low relative expression levels for most of the OBPs across the developmental stages (Fig. 6B). *SiOBP*s still moderately expressed during development (after normalization) were few and included *SiOBP*s 7, 9, and 15 seen in early and late instar larvae, and *SiOBP*s 1, 10 (relatively highly expressed), and 15 in pupae.

### Some evolutionarily related sub-groups of *SiOBP*s show similar expression patterns

**Figure 7:**
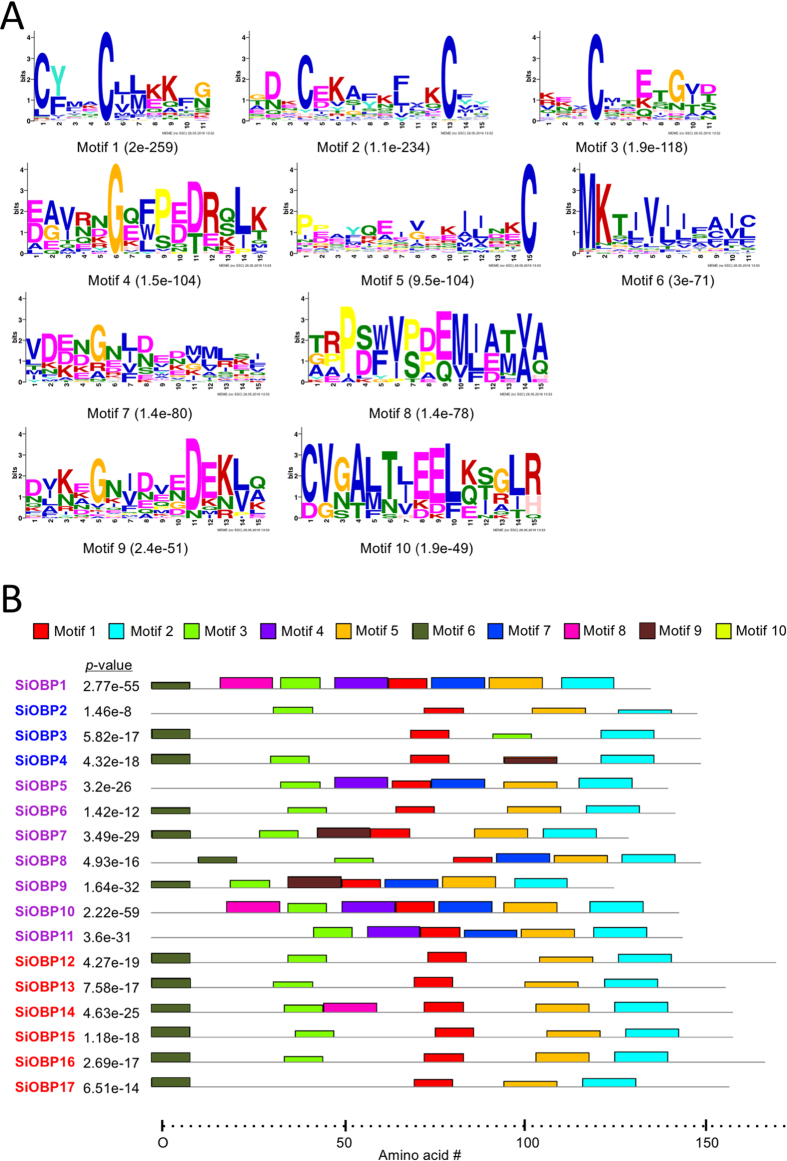
Motif analyses of OBPs. (**A**) Ten most common motifs identified in the 82 OBPs using MEME (version 4.11.2, http://meme-suite.org/tools/meme). The size (height) of the amino acid represents the degree of conservation of that amino acid in the consensus sequence. (**B**) Positions of indicated motifs in the amino acid sequences of the *SiOBP*s. Colored box height is proportional to match to consensus sequence. Motifs 1, 3, 6, and 9 are further annotated in the figure with corresponding numbers and arrows to better distinguish their identities and positions in each sequence. The complete analyses of the 82 OBPs is given in Supplemental Figure S1.

Although no overall relationship between the *SiOBP* phylogenetic groups and expression was seen, certain *SiOBP*s in some phylogenetic subgroups showed similar patterns of expression (Supplemental Excel File S2, these data are identical to the data shown in Figs 3, 4, 5, 6, 7 and Supplemental Excel File S1, but the order of the *SiOBP*s has been rearranged to match the tree topology). These analyses revealed a number of patterns or relationships between *SiOBP* expression and their phylogenetic relatedness. In terms of absolute expression and somewhat clearer when normalized to EF1α expression, *SiOBP*s 12–16 were expressed in a similar manner across castes (male and female alates), developmental stages, and tissues, although some differences were noted in workers where expression of *SiOBP*s 14 and 15 were dissimilar from the rest (Supplemental Excel File S2). SiBOPs 8 and 9, but not 7, were also similarly expressed across caste and tissue, but not in the developmental stages. A pattern was seen between *SiOBP*s 5, 6, and 11 in workers, female alates, and in the developmental stages, but not in male alates, where only *SiOBP*s 6 and 11 were similar in expression. For the subgroup comprised of *SiOBP*s 2, 3, and 4, with the exception of *SiOBP*3 (*Gp-9*), which was highly expressed in all castes, similar trends in expression were noted.

When the data were normalized in terms of tissue distribution, several patterns of *SiOBP* expression emerged (Supplemental Excel File S2, “Tissue distribution %” subtab). In workers, *SiOBP* subgroup 5, 6, and 11 were preferentially expressed in the antennae, whereas *SiOBP*s 2, 3, and 4 and 12–16 (with the exception of SIOBP 15) were preferentially expressed in the head. These patterns were not seen in the male alate, but instead various subgroup preferences, although not containing all members were seen in the abdomen expression profiles. Patterns could also be seen in the female alates, notably with SiBOPs 5, 6, and 11 in the antennae, *SiOBP*s 8, 9 (but not 7), 3, 4 (but not 2), and 12, 13, 16 (but not 14 and 15) preferentially found in the female abdomen.

To confirm differential expression across tissues and across castes/developmental stages, four statistical analyses were performed, namely; (1) comparison of the expression of each *SiOBP* across different tissues (Supplemental Excel File S3), (2) comparison of the expression of different *SiOBP*s within each tissue (Supplemental Excel File S4), and (3) comparison of the total expression of each *SiOBP*s across tissues within each caste (Supplemental Excel File S5). These analyses showed significant (1) differential expression of OBPs within the same tissues, (2) differential expression of individual OBPs across tissues, (3) differential expression of individual as well as the entire set of OBPs across castes, and (4) differential expression of OBPs within developmental stages and between developmental stages as well as between the adult stages.

### Protein motifs identified in the *S. invicta* OBPs

The MEME server^21^ was used to identify conserved amino acid motifs found in the 17 *S. invicta* OBPs, along with the other homologs used to build the phylogenetic tree (82 OBPs total). In total 16 motif with e-values <2e^−15^ were identified, with the 10 most common motif patterns, i.e. those with the lowest e-values and ascending, and their distribution within the *S. invicta* OBPs shown (Fig. 7A,B, respectively). Of the remaining 6 motifs most were not found in the SiOBPs and were thus excluded from the analyses. Motif 10 was not found in any of the SiOBPs, but was amongst the top 10 most common motifs due to its presence in the other OBPs examined. The full distribution of the 10 most common motifs across all of the OBPs examined is given in Supplemental Fig. S1. SiOBP8 contained 8 out of the top 10 most common motifs, whereas SiOBPs 2, 3, and 17 contained only 4 out the top 10 motifs identified. Motifs 1 and 2 were in all SiOBPs, although divergence of these motifs was seen in SiOBP2 (for both motifs 1 and 2; the height of color blocks in figure is proportional to the e-value confidence, i.e. the level in which the sequence highlighted is identical/similar to the consensus sequence, thus a sequence in which the motif is represented by a smaller block indicates that it has diverged the consensus), and SiOBP8 (for motif 1 only). Motifs 3 and 5 were found in all SiOBPs with the exception of motif 3 not found in SiOBP17 and motif 5 not found in SiOBP3. Motif divergence was seen in SiOBP2 (motifs 3 and 5), SiOBPs 3, 6, 8, 13, 15, and 16 (motif 3), and in addition, the motif 3 found in *SiOBP*3 was notably in a different position (towards the C-terminus) as compared to its position in the other SiOBPs. Motif 4 was found only in SiOBPs 1, 5, 10, and 11, whereas motif 6 was found in most SiOBPs, being absent in SiOBPs 2, 5, and 11. As this latter motif (6) was localized to the N-terminus (except for SiOBP8 which as shifted slightly towards the C-terminus), it likely reflects signal peptide similarities. Motif 7 was identified in SiOBPs 1, 5, and 8–11, whereas motif 8 was found only in SiOBPs 1, 10, and 14. Motif 9 was found in SiOBPs 4, 7, and 9, although the sequence had somewhat diverged in SiOBP4.

## Discussion

The generalized model of insect chemosensation involves a suite of proteins that function to relay identification of chemical ligands to neurophysiological and behavioral responses. Within the context of the initial detection of chemical molecules, the major proteins involved are thought to include sensory neuron membrane proteins (SNMPs), odorant and ionotropic receptors (ORs and IRs, respectively), gustatory receptors (GRs), the soluble odorant binding and chemosensory proteins (OBPs and CSPs), and odorant-degrading enzymes (ODEs), all housed within specialized sensilla that are found, although not exclusively, on the antennae^2^. The water soluble CSPs and OBPs act as ligand binding proteins to capture often hydrophobic compounds, e.g. environmental volatiles and pheromones, transporting them to the neuronal membrane receptors. In all insects examined thus far, CSPs and OBPs, exist as highly divergent gene families, and *S. invicta* appears to have one of the smaller sets of OBPs (i.e. 17–18) as compared to fruit flies, (*Drosophila melanogaster*; 61), mosquitoes (*Aedes* sp.; 64–72 and *Culex quiquefasciatus*; 53), although the honey bee (*A. mellifera*; 21), the tsetse fly (*Glossina mortsitans*; 20), and the pea aphid (*Acyrthosiphon pisum*; 15) have similar numbers of *OBP*s in their genomes^22^. The best studied of these are the Lepidopteran proteins where significant information concerning distribution, expression, pheromone and ligand binding, and structural determinations have been made^22^,^23^,^24^,^25^,^26^,^27^,^28^. Genomics analyses in Lepidoptera have indicated intriguing possibilities of OBP gene loss and gain linked to behavioral shifts. For example, butterflies appear to have lost a specific OBP involved in moth pheromone detection, which the authors suggest may correlate to shifts in stimulus perception/modes of communication from olfactory in moths to visual in butterflies^29^. Expression (transcriptomic) studies have been the main source of OBP discovery including in the tobacco cutworm, *Spodoptera litura*, the tsetsefly, *Glossina mortsitans*, winged and unwinged morphotypes of the pea aphid, and several social hymenoptera, amongst others^30^,^31^,^32^,^33^.

Although their names, i.e. OBPs and CSPs, suggest functions in olfaction, it has long been recognized that these proteins do not function exclusively in the antennae of insects and are likely involved in many processes aside from olfaction^4^,^9^. Indeed, current ideas suggest that only a subset of OBPs are directly involved in olfaction, notable amongst these being the pheromone binding proteins (PBPs, considered a sub-group within the OBPs). However, the high degree of divergence seen in OBPs renders extrapolation of results from one insect species to another difficult. A comparative analysis of the *S. invicta* OBPs with that of the honeybee, *A. mellifera* (and as partially recapitulated here), revealed several branch point separations of the OBPs into distinct groups^16^. These analyses indicate at least two distinct OBP groupings; SiOBPs 1, 5, 6, and 9–11 are found in one group and have conserved shared orthologs with various ant OBPs. SiOBPs 2–4, 7, 8, and 12–17 are found within a second grouping, with SiOBPs 2, 3, and 4 having conserved *A. mellifera* orthologs, and SiOBPs 12–17 appearing to represent an *S. invicta*-gene expansion, with one shared homolog in the ant, *A. echinatior*. Interspersed within the second overall grouping there exists a divergent *Apis* spp.-specific OBP gene expansion (AmOBPs 13–20), with SiOBPs 7 and 8, the most closely related to these. With respect to the bee OBPs, *AmOBP*s 13–21 are found as a tandem genomic array with AmOBPs 14–21 designated as C-minus OBPs due to the absence of two of the six usually conserved cysteine residues^34^. With the exception of *SiOBP*14, the other members of the fire ant expansion were found on the same linkage group contig (lg16), along with *SiOBP*s 3, 4, 5, and 9.

Protein amino acid sequence analyses of 82 OBPs related to the *S. invicta* repertoire allowed for the identification of a set of common motifs. The highly conserved cysteine residues, characteristic of OBPs were found in motifs 1, 2, 3, and 5, and these corresponded to the most widely conserved motifs found in most of the SiOBPs. However, in a number of cases a specific motif appears to have been lost in a particular lineage, e.g. motif 3 in SiOBP17, divergence of motifs 2 and 5 in SiOBP2. Motif 6, while not part of the conserved cysteine containing motifs, was also widely distributed, likely because it corresponds to a conserved signal peptide processing sequence. However, this motif was absent in SiOBPs 2, 5, 10, and 11, and while these latter proteins also contain signal sequences, these appear to have diverged significantly enough that they no longer resemble the others. As expected, the distribution of many motifs mirrored the phylogenetic tree, although a few notable exceptions were seen. SiOBPs 3 and 4, although very similar in their exon-intron structure and phylogenetic placement, showed differing numbers and organization of motifs. SiBOP14 while closely sharing related motifs found in SiOBP2 12, 13, 15–17, also contained one motif (8) found only in SiOBPs 1 and 10 (amongst the SiOBPs). Similarly, SiOBPs 7 and 9, shared a common motif (9) not present in the other SiOBPs. Some of these motifs may contribute to either substrate binding and/or specificity or to interactions with downstream partners, e.g. receptors, whether odorant or otherwise.

Our data indicate, that in fire ant workers, while a distinct subset of *SiOBP*s (*SiOBP*s 1,2, 5,6, 11> *SiOBP*s 7, 10, and 15) are expressed in the antennae, higher levels are generally seen in worker heads, somewhat less in the thorax, and notably lower in the abdomen. *SiOBP* expression in the antennae of female fire ant alates was similar to workers, whereas expression in male alate antennae was noticeably lower. The low absolute expression levels of the OBPs seen in the antennae are in general agreement with observations of the low incidence of OBPs in ant antennae and the suggestion that ant olfaction relies more on the functions of CSPs^35^,^36^,^37^ or other proteins, an example of the latter being an apolipophorin-like protein identified as being expressed in *S. invicta* antennae^38^. This conclusion also agrees with expression analysis of the OBP repertoire in *A. mellifera* that indicate that OBPs are broadly expressed, and indeed, may only play a minor role in olfaction in some insects^34^. However, some caution should be taken in this interpretation in that particularly in workers, normalization to EF1α levels indicates robust expression of a subset of *SiOBP*s in the antennae. This analysis is consistent with a recent transcriptomics study of several ant species including *Cerapachys biroi*, *Camponatus floridanus*, and *Harpegnathos saltator*, which indicated that subsets of both CSPs and OBPs are expressed in the antennae, suggesting that CSPs have not replaced OBPs in ant olfaction^8^. After normalization, *SiOBP*s 1, 2, 5 and 6 showed the highest relative expression levels in antennae across all castes, with no strongly caste-specific *SiOBP* expression seen. Low expression of *SiOBP*s 4, 8, and 9 was seen in all antennae regardless of caste. Mass spectrometry analyses have indicated that *SiOBP*s 2 and 15, both of which our data indicate are moderately expressed in these tissues, can be found in the antennae of *S. invicta* workers^16^,^37^, leaving open the possibility of some *SiOBP*s playing a role in antennal chemical compound ligand binding.

A significant literature exists regarding *SiOBP*3 (*Gp-9*), originally described as a “social organization gene” due to the observation of distinct alleles that correlated with whether colonies were monogyne (single queen) or polygyne (multiple queens) in nature^13^,^14^,^39^. However, the link between *SiOBP*3 and sociality has been met with some controversy^17^,^18^. Recent evidence suggests that this gene is part of a much larger chromosomal segment of ~13 Mb, comprising of more than half the chromosome and containing >600 ORFs, that has undergone distinct rearrangement events in the two alternate *S. invicta* social forms (mongyne versus polygyne) and whose recombination is suppressed^19^. As noted above, this region (corresponding to linkage group 16) contains not only *SiOBP*3, but also *SiOBP*s 4, 5, 9, 12, 13, and 15–17, thus significantly expanding the range of chemical compounds and the OBPs that may play a role in mediating this specific aspect of social organization. Our data show robust expression of *SiOBP*3 in workers, and male and female alates, with very high expression in worker heads, and throughout female alates. Interestingly, relative expression was lowest in antennae regardless of caste and low expression of *SiOBP*3 was seen throughout the developmental stages examined, i.e. eggs, larvae, and pupae.

Each caste showed a distinct overall *SiOBP* expression pattern, with the differential expression of a number of specific proteins. *SiOBP*s were generally preferentially expressed in male and female alates throughout the body, i.e. head, thorax, and abdomen, as compared to the antennae. In contrast, *SiOBP* expression was generally lower in the abdomen (and antennae) of workers, and more robustly expressed in the head and thorax. These data suggest generalized roles for most *SiOBP*s and the likelihood that a number of these proteins are hemolymph proteins that function as ligand carriers of endogenous ligands, e.g. hormones and signaling molecules. A number of *SiOBP*s were expressed in a sex-specific manner. Expression of *SiOBP*4 in the thorax of female alates was very high, whereas neither workers nor male alates expressed this gene at such high levels. *SiOBP*4 was also notably absent in antennal fractions. Conversely, *SiOBP*9 was highly expressed in the male alate abdomen, but noticeably absent in both female alates and workers. *SiOBP*s 13, 15, and 16 were noticeably more highly expressed in worker heads than female and male alate counterparts, and *SiOBP*14 was highly expressed in the thoraces of workers but expressed at moderate to low levels in female and male alates, respectively.

In terms of absolute expression levels, a number of *SiOBP*s were expressed a moderate levels during the various developmental stages examined, indicating that these proteins may have some functioning during development. It should be noted that no attempt was made to discriminate between whether the larvae corresponded to workers or male/female alates visual inspection of the colony indicated less than 2% winged alates, and it is likely that the majority of the larvae examined corresponded to workers. With respect to the developmental stages examined, and in contrast to adult tissues, normalization to EF1α levels had a significant effect on the relative expression levels of the *SiOBP*s, primarily due to the fact that EF1α levels were very high in these samples. These analyses revealed low relative expression of most *SiOBP*s across the developmental stages examined. Notable exceptions included *SiOBP*1 found during all the developmental stages, *SiOBP*s 8, 10, and 15, found in early and late instars, and *SiOBP*s 10 (highest expressed *SiOBP* detected) and 15 in pupae. These data indicate the potential for some OBPs to function as important developmentally linked protein. Such a finding has been seen for a number of CSPs, e.g. AmCSP5 and SiCSP9, implicated in mediating embryonic development in *A. mellifera* and larval molting in *S. invicta*, respectively^40^,^41^. It is tempting to speculate that some OBPs act as carriers of developmental signaling molecules and further studies probing such functions are warranted. The distribution of OBPs beyond antennal and during developmental, i.e. larval and pupal stages, has also been reported for the parasitoid wasp, *Sclerodermus* sp., although it is not clear whether the full set of these proteins in this insect has been identified^5^. These data do support a model in which OBPs are likely general binding proteins, with only a subset involved in antennal binding of ligands.

A subset of *D. melanogaster* OBPs have been functional examined using RNAi mediated knockdown^42^. *SiOBP*s 2–4 and 12–17 appear to be orthologs of the *D. melanogaster* DmOBP59a, an OBP whose expression while not readily detectable in fruit fly antennal extracts, appears to mediate altered behavior responses to a broad range of odorants. SiBOPs 1 an 10 were similar to DmOBP83a, an OBP expressed in fly antennal extracts and appears to mediate behavioral responses to a narrow range of chemical odorants including citral, acetophenone, *I*-carvone, and 2-heptanone^42^. Notably, whereas *SiOBP*1 is expressed in *S. invicta* antennae, particularly in workers, *SiOBP*10 is expressed in the head (all castes) and during development (larvae and pupae). *SiOBP*s 7 and 8, correspond to the DmOBP99b/83c/56a family, two of which (56a and 99b) are robustly expressed in *D. melanogaster* antennal extracts. DmOBPs 56a and 83c appear to mediate responses to a small number of odorants in a sex-specific manner, whereas DmOBP99b RNAi knockdowns displayed a broader altered response pattern^42^. Neither *SiOBP*7 nor 8 were strongly expressed in ant antennae; however, *SiOBP*7 was very highly expressed in worker heads and throughout the bodies of male and female alates. *SiOBP*8, however, was poorly expressed in most tissues examined. In *Drosophila*, RNAi knockdown also resulted in altered antennal electrophysiological responses for several of the OBPs examined, an effect also seen for an OBP (OBP2) in the cotton aphid, *A. gossypii*^43^.

We have conducted a systematic expression profiling of the *SiOBP* gene family in a eusocial insect. Patterns of *SiOBP* gene expressed were found to show some relationships to their evolutionary relatedness, i.e. phylogeny, although no absolute overall correlation was observed. Preferential expression of phylogenetically related *SiOBP*s subgroups were seen that was caste dependent, whereas others showed tissue and/or developmental preferences, although normalized expression during development was very low for most *SiOBP*s. The small size of the fire ant OBP-gene family and their relatively low expression in antennae suggest that these proteins may only be nominally involved in direct olfaction and/or olfactory coding. Instead, our data support a model in which these proteins act as ligand carriers throughout the body, some likely functioning to carry specific molecules mediating specific processes, whereas others may be more general in nature. This does not preclude the participation of individual OBPs in aspects of olfaction, e.g. by binding and/or sequestering endogenously produced pheromones or other semiochemicals and/or ultimately acting during their release. However, our data do indicate that the term “odorant-binding protein” is likely to be an unfortunate designation for many of these proteins and that a more generic term such as “ligand-carrier proteins” would be more apt. Our data provide a basis for future work aimed towards better understanding the diverse functions of OBPs. Determining the chemical compounds bound by the OBPs and the protein targets and/or interacting partners of these proteins, e.g. membrane receptors, is needed to further characterize the physiological role(s) of the *SiOBP*s.

## Methods

### Insects and experimental samples

*S. invicta* colonies were collected from the field and maintained in Fluon-coated and/or talcum powder dusted trays as described^44^. Three independent colonies were sampled. Colonies were assessed to be polygyne due to the presence of multiple-queens in the founding colony and via sequencing of the full-length cDNA of *SiOBP3/Gp-9* in which both *Gp-9B* and *Gp-9b* alleles were detected. Colonies were fed with 300 mM sucrose and freeze dried *Galleria mellonella* larvae and kept at room temperature with ~70% humidity and 16:8 dark:light photoperiod. For each colony, around 200–500 workers, males and female alates were collected separately, immersed in RNALater (Invitrogen, Thermo Fisher Scientific, Waltham, MA) and dissected under a stereomicroscope into four sections; antenna, head (without antennae), thorax and abdomen. Approximately 200–500 larvae and pupae collected for the various development stages. Eggs were collected from mated queens collected from colonies within 24 hours and samples were immediately suspended in RNALater and stored at −80 °C until RNA extraction. Similarly, small larvae, large larvae and pupa were collected and immersed in RNALater before RNA extractions. Adult stages were not sampled as same-age cohorts and larvae were distinguished by their size only and likely reflect a mixture of minor and major workers, as well as potential male and female alates. Early instars (1^st^ and 2^nd^) were selected based on size and color with early instars (1^st^ and 2^nd^) displaying some melanization and late (3^rd^ and 4^th^) clear to whitish. Generally the early instars were smaller than 1 mm (≤1 mm), and late instars (3^rd^ and 4^th^) were between 1.1 and 4.5 mm.

### RNA preparation and cDNA library construction

Samples (~100 mg) were ground in liquid nitrogen and then mixed with Trizol reagent (1 ml) and total RNA was extracted following the manufacture’s instructions (Invitrogen, Life Sciences). Genomic DNA in samples was digested using TURBODNase (Invitrogen) according to the manufacture’s instruction. Total RNA quality and quantity were analyzed by agarose gel electrophoresis and via NanoDrop 2000 spectrophotometric analyses. Quantification of RNA concentrations in samples was performed using a Qubit H 2.0 fluorometer (Invitrogen, Carlsbad, CA). Finally, 2 μg total RNA from different tissues were used to construct cDNA libraries using High-Capacity cDNA Reverse Transcription Kit (Applied Biosystems, Foster City, CA) in terms of standard protocol.

### Absolute qualification of cDNAs

The sequences of the *S. invicta* odorant binding proteins (*SiOBP*s) were downloaded from NCBI (Supplemental Table S1). Absolute qualification of all OBPs was performed as previous described^45^. Briefly, specific qRT-PCR primers were designed using Beacon designer 8.13 (Palo Alto, CA, USA) and synthesized by Invitrogen Ltd (Carlsbad, CA). The list of primers used is given in Supplemental Table S2. Primers were used to amplify the respective regions of the *SiOBP*s using an *S. invicta* cDNA library as described above as the template. After purification, the PCR generated fragments were ligated into the pGEMT vector (Promega Corp., Madison, WI). Positive clones were identified by colony PCR and plasmids were isolated and the integrity of the inserts verified by sequencing (Eton Biosciences, San Diego, CA). Plasmid concentrations were quantified using Qubit dsDNA BR Assay Kit (Invitrogen, Carlsbad, CA). RT-PCR primer sets were validated for amplicon size, optimal ratios, T_m_, and efficiency (Supplemental Table S3). The amplification efficiency (E) was calculated using the slope of a linear regression determined by the Ct values (axe Y) and the concentration of cDNA in logarithmic scale (axe X). Given the slope value, the Efficiency (E) value was calculated using the formula:


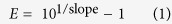


Plasmid constructs and optimized the T_m_ values were used to construct expression standard curves, using serial dilution of the plasmid templates (10^−5^ to 10^−9^). The number of transcript copies was calculated using the molecular weight of plasmids and their empirically determined concentrations using the following formula:


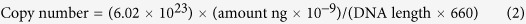


The standard curves and empirically determined C_t_ values as derived from the q-RT-PCR experiments were used to calculate the absolute transcript expression values for the OBPs in the various samples. For qRT-PCR, the 2x SYBR Green qPCR Master Mix (Biotools, Houston TX) was used for the reactions. *S. invicta* cDNA libraries from different tissues were diluted 40 times and 5 μL of diluted cDNA were used as template in a 15 μL reaction volume. Each reaction included 1 × Master Mix, 5 μL template and 200 nM of each of gene specific primer pairs. Real time q-PCR reactions were performed for at least two RNA preparations from each tissue sample. Controls and internal references included primers designed to the EF1α and GAPDH genes of *S. invicta* that were used for initial normalization of the template amount and whose absolute copy numbers were also quantified. The PCR reactions were performed using a Eco Real-Time qPCR System (Illumina, San Diego, CA) with a thermo-profile of one cycle of 95 °C 5 min, 95 °C 2 min, then 45 cycles of 95 °C 15 s, and 60/59 °C 45 s, followed by a melting curve analysis from 55 to 95 °C.

### Data analyses

An analysis of variance (ANOVA) was conducted to evaluate potential significant differences in expression levels among tissues and castes/developmental stages. The data, number of copies per μg of RNA, were transformed logarithmically in order to correct normality and unequal variances. Means were compared with LSD (Least significant differences) test and the Duncan’s new multiple range test (MRT). Statistical analyses were performed using the IBM SPSS Statistics, version 21 (Armonk, NY).

### Phylogenetic and motif analyses

A total of 82 OBPs from *S. invicta*, *A. echinatior*, *C. floridanus*, and *H. saltator*, *A. mellifera* and *A. cerana* were used for phylogenetic tree construction and motif analyses (Supplemental Table S4). Putative N-terminal signal peptides were identified using Signal P (http://www.cbs.dtu.dk/services/SignalP/). Exon-intron splice positions were identified using the online Gene Structure Display Server (GSDS: http://gsds.cbi. pku.edu.cn). Amino acid motifs were identified using MEME (version 4.11.2, http://meme-suite.org/tools/meme). Amino acid multiple sequence alignments (MSAs) were generated with five different alignment algorithms: MUSCLE, PRANK, MAFFT, CLUSTALW and PROBCONS^46^,^47^,^48^,^49^,^50^. For each MSA the best fitting model of amino acid substitution was estimated with MEGA 6.0^51^. MEGA optimizes the tree topology search starting with a Neighbor Joining tree and uses the likelihood function and three model criteria BIC (Bayesian Information Criterion), AIC (Akaike information criterion) and LnL (log likelihood) to find the best fitting model of amino acid substitution. MEGA consistently chose the model LG + G + I with 5 rates categories (G = Gamma shape parameters and I = proportion of invariant sites). The different amino acid MSAs were analyses with PhyML 3.0 available at the phylogeny.fr platform^52^,^53^ to see the alignment method yielding the most likely tree (i.e. ability to best capture phylogenetic signal) given the LG amino acid substitution model and estimating G and I^54^. The PRANK algorithm gave the best tree (lowest LnL, parsimony score and tree size), which was re-built using RaxML at the CIPRES Science Gateway^55^,^56^, and implementing the LG amino acid substitution model with 5 rates of categories. G and I were estimated, branch lengths optimized and branch support calculated by bootstrapping. RaxML was allowed to halt bootstrapping automatically. The software MEGA 6.0 was used to draw the tree.

## Additional Information

**How to cite this article**: Zhang, W. *et al*. Tissue, developmental, and caste-specific expression of odorant binding proteins in a eusocial insect, the red imported fire ant, *Solenopsis invicta*. *Sci. Rep.* **6**, 35452; doi: 10.1038/srep35452 (2016).

## Supplementary Material

Supplemental Information

Supplementary Excel File S1

Supplementary Excel File S2

Supplementary Excel File S3

Supplementary Excel File S4

Supplementary Excel File S5
