# Supplementary material for: Tissue, developmental, and caste-specific expression of odorant binding proteins in a eusocial insect, the red imported fire ant, Solenopsis invicta
Source: Sci Rep. 2016 Oct 21;6:35452. doi: 10.1038/srep35452 (PMC5073229; doi:10.1038/srep35452)
Supplement: Supplemental Information [file srep35452-s1.pdf]

Supplemental Materials

Tissue, developmental, and caste-specific expression of odorant binding proteins in a eusocial insect, the red imported fire ant, *Solenopsis invicta*

Wei Zhang, Arun Wanchoo, Almudena Ortiz-Urquiza, Yuxian Xia, and Nemat O. Keyhani

Supplemental Tables

Table S1. List of *S. invicta* OBP genes

Name	AA Length	GenBank accession number	Cysteine	Signal Peptide
SiOBP1	139	HQ853350	6	1-19
SiOBP2	152	HQ853351	4	1-17
SiOBP3	153	AF427898.1	6	1-19
SiOBP4	153	HQ853352	6	1-19
SiOBP5	144	HQ853353	6	1-22
SiOBP6	146	HQ853354	6	1-23
SiOBP7	133	HQ853355	6	1-17
SiOBP8	153	HQ853356	4	No SP
SiOBP9	129	HQ853357	6	1-20
SiOBP10	147	HQ853358	6	1-19
SiOBP11	149	HQ853359	6	1-24
SiOBP12	174	HQ853360	6	1-20
SiOBP13	160	HQ853361	6	1-18
SiOBP14	162	HQ853362	6	1-19
SiOBP15	162	HQ853363	6	1-22
SiOBP16	171	HQ853364	6	1-20

Table S2. List of primers used for quantitative RT-PCR primers

	Forward	Reverse
SiOBP1	TGACCGGTCTATCACGTGCT	GACGCATTTCGCCACATTGT
SiOBP2	TCATTGAAAGAGTGGGCCGAAT	GCAGTCAGGCATTCCGTTAGA
SiOBP3	TGAAGATACCGAACTACACAA	AGCACCTGTTTCCTTGAT
SiOBP4	AGCTCTTCTCGGTTGGTGAA	GCCCAAGCTTTTGTCACATT
SiOBP5	ACGCATAGATGACGGTAT	TGATAAGTGGTTGAGCAATC
SiOBP6	ATATTTGCCAGTTGTCGTAAA	ATCGTTGGATTAACATCATAGAG
SiOBP7	AAAGGAGAGGTAGCTGAAAGTGAC	TGCCTCCTTTTTCACAACCTGT
SiOBP8	TTAAGAGGCTACAGAACATTATG	AGAGTTACCTTCCAGTCAAT
SiOBP9	AGGCTGGAAACATTGAACAAAA	TATCGCATGCATTCTCCGCT

SiOBP10	TTGGTCTGGTCGACGACAAG	GGAGTAGCTGCGCGTAACA
SiOBP11	TTCTCGTCTGTGTCTCTG	TCTGCTCCTTCTAACTTATCA
SiOBP12	ATGGAAGGAACCAACATTAAAG	CCTGCATACATTTTCTTTCTTC
SiOBP13	GCTGTGCGCATGTGTTTTGA	ACACGCCATAAAGCAGCCAT
SiOBP14	CCCACCAGCCGAAATAATAGCT	CGCAGCCGCTTCTTTTACG
SiOBP15	GCTGACTTTGTCAAACCGATGG	AATTTGGTGCATTGGTCCGC
SiOBP16	AAAAGTCTCGTGCTTTGCGC	TGCAACAAAGCAACCAACT
SiOBP17	TGCTTTGTGCCTGTGTTTTA	TTTCTGCATTCACAGATTCA
EF1-α	AAGAGAACCCGAAAGCCATT	GCCTCAACGCACATAGGTTT
GAPDH	AAGCTGTGGCGTGATGGCCG	AGGAGGCAGGCTTGGCGAGT

Table S3. Validation of RT-PCR Primer sets.

Gene	Amplicon size (bp)	Exon Location	Approximate size on gel (bp)[1]	Optimal ratio (μM:μM)	T_M (°C)	Efficiency (%)
SiOBP1	202	183-384	200	2:2	59	106.32
SiOBP2	218	154-371	200	2:2	60	93.54
SiOBP3	195	189-315	200	2:2	59	105.57
SiOBP4	263	140-402	250	2:2	60	90.16
SiOBP5	135	249-383	150	2:2	60	102.03
SiOBP6	120	306-425	120	2:2	60	110.41
SiOBP7	205	148-352	200	2:2	58	100.51
SiOBP8	185	142-326	200	1.5:1.5	58	97.76
SiOBP9	198	122-319	200	2:2	60	105.80
SiOBP10	190	233-422	190	2:2	59	93.08
SiOBP11	175	20-194	180	**2:2**	60	95.75
SiOBP12	222	268-469	220	2:2	60	95.06
SiOBP13	220	15-234	220	2:2	60	94.30

SiOBP14	289	159-447	300	1.5:1.5	60	100.32
SiOBP15	207	157-363	200	2:2	60	101.08
SiOBP16	237	4-240	250	1.5:1.5	60	103.00
SiOBP17	198	14-211				
EF1-α	79		100	1.5:1.5	60	108.24
GAPDH	184		200	2:2	60	100.35

[1]Determined by agarose gel electrophoresis.

[2]Primers failed to prime against RNA samples and did not produce usable E-values (less than 90% efficiency).

Table S4. List of proteins used in phylogenetic analyses (Fig. 1)

Species	Name	NCBI Reference Sequence
Harpegnathos saltator	HsOBP1-X1	XP_011144088
	HsOBP1-X2	EFN89245
	HsOBP2	EFN79108
	HsOBP3	XP_011139553
	HsOBP4	XP_011147605
	HsOBP5-X1	EFN77138
	HsOBP5-X2	XP_011150623
	HsOBP6	EFN84434
	HsOBP7	XP_011150624
	HsOBP8-X1	XP_011145649
	HsOBP8-X2	EFN80433
Camponotus floridanos	CfOBP1	XP_011251750
	CfOBP2	XP_011254774
	CfOBP3	EFN68669
	CfOBP4	XP_011268025

	CfOBP5-X1	XP_011253322
	CfOBP5-X2	XP_011253323
	CfOBP6	XP_011253326
	CfOBP7	XP_011255852
Acromyrmex echinatior	AeOBP1	EGI67501
	AeOBP2	EGI61877
	AeOBP3	EGI63313
	AeOBP4	EGI61878
	AeOBP4	EGI61878
	AeOBP5	EGI63909
	AeOBP6	XP_011056723
	AeOBP7	EGI57538
	AeOBP8	EGI63317
	AeOBP9	XP_011069000
	AeOBP10	XP_011069001
	AeOBP11	XP_011068667
	AeOBP4	EGI61878
	AeOBP5	EGI63909
	AeOBP6	XP_011056723
	AeOBP7	EGI57538
	AeOBP8	EGI63317
	AeOBP9	XP_011069000
	AeOBP10	XP_011069001
	AeOBP11	XP_011068667
Solenopsis invicta	SiOBP1	NP_001291522
	SiOBP2	ADX94399
	SiOBP3	XP_011157711
	SiOBP4	XP_011157725
	SiOBP5	XP_011156042

	SiOBP6	XP_011165204
	SiOBP7	XP_011167532
	SiOBP8	XP_011170254
	SiOBP9	XP_011173007
	SiOBP10	XP_011171270
	SiOBP11	XP_011171271
	SiOBP12	ADX94408
	SiOBP13	XP_011157738
	SiOBP14	ADX94410
	SiOBP15	ADX94411
	SiOBP16	ADX94412
Apis cerana	AcOBP1	AEZ65021
	AcOBP2	ABD97847
	AcOBP3	AHN15444
	AcOBP4	AKQ98505
	AcOBP5	AAR83081
	AcOBP6	ABD97844
	AcOBP10	AKQ98506
	AcOBP11	AGQ03796
	AcOBP12	ALR87119
	AcOBP13	ALR87120
	AcOBP14	AKQ98508
	AcOBP15	ALR87121
	AcOBP17	AJA33390
	AcOBP21	AKQ98509
Apis mellifera	AmOBP1	NP_001011590
	AmOBP2	NP_001011591
	AmOBP3	NP_001035311
	AmOBP4	NP_001011589

AmOBP5	NP_001011588
AmOBP6	NP_001011593
AmOBP7	NP_001035310
AmOBP8	NP_001164515
AmOBP9	NP_001035315
AmOBP6	NP_001011593
AmOBP7	NP_001035310
AmOBP8	NP_001164515
AmOBP9	NP_001035315
AmOBP10	NP_001035294
AmOBP11	NP_001035316
AmOBP12	NP_001035319
AmOBP13	NP_001035314
AmOBP14	NP_001035313
AmOBP15	NP_001035298
AmOBP16	NP_001035295
AmOBP17	NP_001035297
AmOBP18	NP_001035317
AmOBP19	NP_001035299
AmOBP20	NP_001035312
AmOBP21	NP_001035296

Supplemental Fig. S1

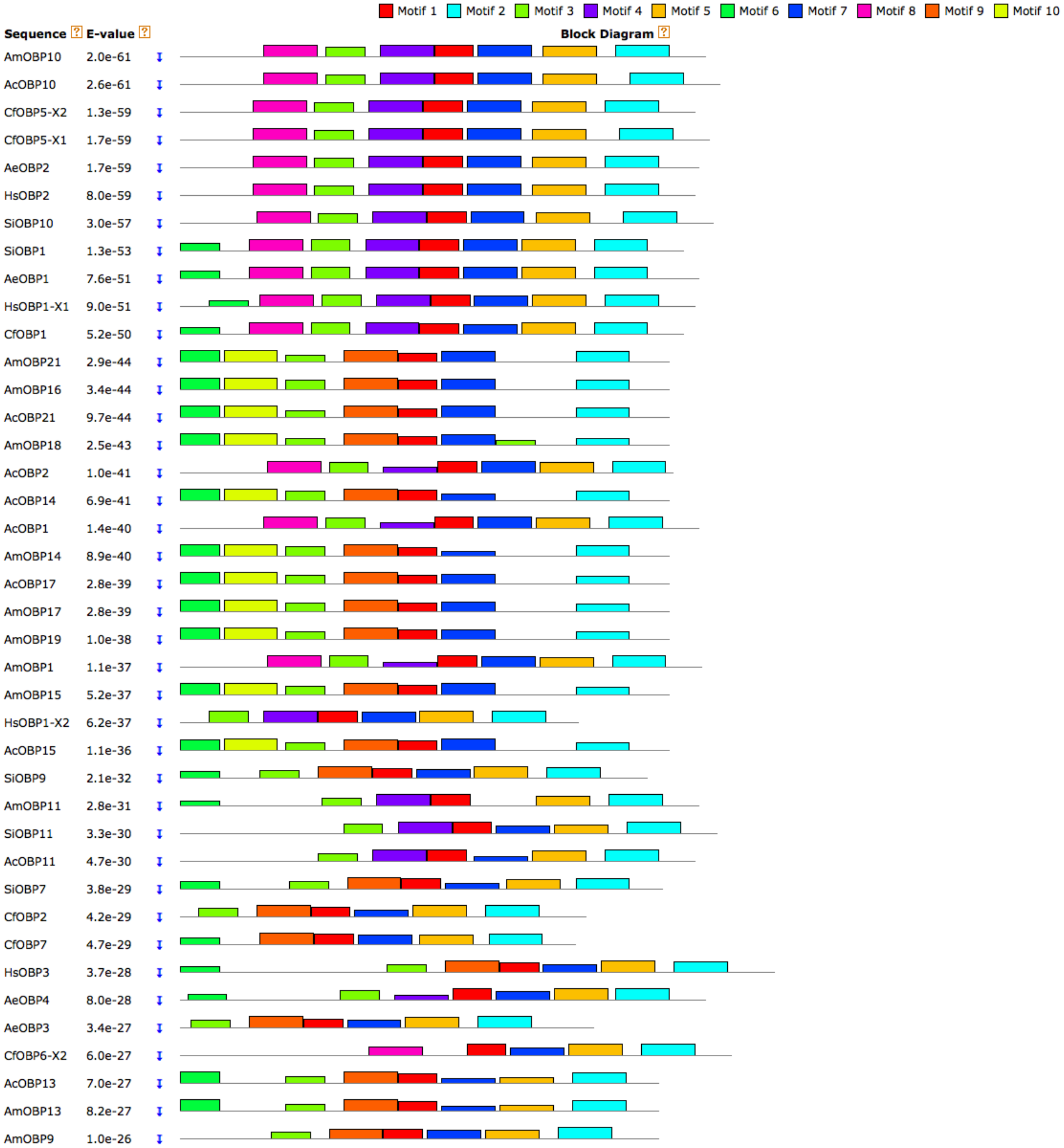

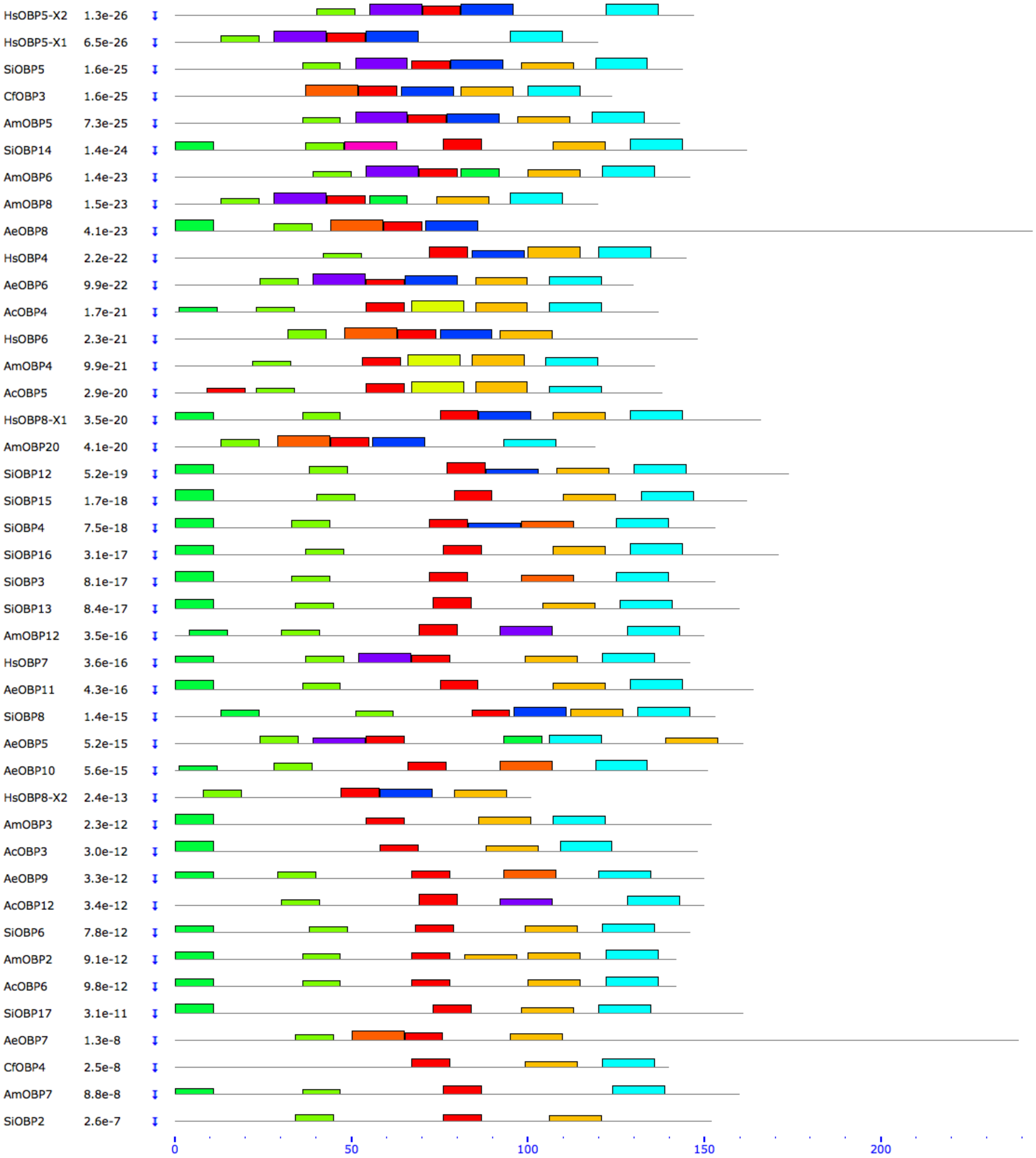
HsOBP5-X2 1.3e-26
HsOBP5-X1 6.5e-26
SiOBP5 1.6e-25
CfOBP3 1.6e-25
AmOBP5 7.3e-25
SiOBP14 1.4e-24
AmOBP6 1.4e-23
AmOBP8 1.5e-23
AeOBP8 4.1e-23
HsOBP4 2.2e-22
AeOBP6 9.9e-22
AcOBP4 1.7e-21
HsOBP6 2.3e-21
AmOBP4 9.9e-21
AcOBP5 2.9e-20
HsOBP8-X1 3.5e-20
AmOBP20 4.1e-20
SiOBP12 5.2e-19
SiOBP15 1.7e-18
SiOBP4 7.5e-18
SiOBP16 3.1e-17
SiOBP3 8.1e-17
SiOBP13 8.4e-17
AmOBP12 3.5e-16
HsOBP7 3.6e-16
AeOBP11 4.3e-16
SiOBP8 1.4e-15
AeOBP5 5.2e-15
AeOBP10 5.6e-15
HsOBP8-X2 2.4e-13
AmOBP3 2.3e-12
AcOBP3 3.0e-12
AeOBP9 3.3e-12
AcOBP12 3.4e-12
SiOBP6 7.8e-12
AmOBP2 9.1e-12
AcOBP6 9.8e-12
SiOBP17 3.1e-11
AeOBP7 1.3e-8
CfOBP4 2.5e-8
AmOBP7 8.8e-8
SiOBP2 2.6e-7
0
50
100
150
200

Supplemental Figure S1 7. Motif analyses of OBPs. Analyses of the 82 OBPs used in the phylogenetic tree (Figure S1 and Supplemental Table S4) using MEME (version 4.11.2, http://meme-suite.org/tools/meme). The size (height) of the amino acid represents the degree of conservation of that amino acid in the consensus sequence.
